# Effect of peramivir on respiratory symptom improvement in patients with influenza virus infection and pre‐existing chronic respiratory disease: Findings of a randomized, open‐label study

**DOI:** 10.1111/irv.12788

**Published:** 2020-07-17

**Authors:** Motokazu Kato, Yutaka Saisho, Hiroshi Tanaka, Takuma Bando

**Affiliations:** ^1^ Chest Disease Clinical and Research Institute Kishiwada City Hospital Osaka Japan; ^2^ Shionogi & Co., Ltd. Osaka Japan; ^3^ Sapporo Cough Asthma and Allergy Center Sapporo Japan; ^4^ Bando Internal Medicine Clinic Hakusan Ishikawa Japan; ^5^ Present address: Respiratory Institute, Kamei Hospital Osaka Japan

**Keywords:** asthma, cough, influenza A virus, influenza B virus, neuraminidase, peramivir, pulmonary diseases, chronic obstructive; signs and symptoms, respiratory

## Abstract

**Background:**

The efficacy of neuraminidase inhibitors on improvement of respiratory symptoms triggered by influenza in patients with pre‐existing chronic respiratory diseases is unknown.

**Methods:**

This 2‐week, randomized, open‐label study evaluated intravenous peramivir 600 mg on two consecutive days (peramivir‐repeat), peramivir 300 mg single dose (peramivir‐single), and oral oseltamivir 75 mg twice daily for 5 days in patients with confirmed influenza and chronic respiratory diseases. Patients recorded symptom scores daily. The primary endpoint of cumulative area of time vs symptoms (CATVS) was expressed as an index value of area under the curve vs time of the total score of cough, sore throat, and nasal congestion from baseline to 2 weeks.

**Results:**

Of 214 randomized patients, 209 (56% female, 77% aged <65 years, 94% outpatients, 91% bronchial asthma, 62% influenza A) received ≥1 dose of study drug. Mean (standard deviation) CATVS was similar for peramivir‐repeat (782.78 [487.17]) vs peramivir‐single (717.35 [347.55]; *P* = .4371), and for peramivir‐repeat vs oseltamivir (856.34 [404.99]; *P* = 1.00). However, CATVS was significantly shorter for peramivir‐single vs oseltamivir, with an estimated treatment difference (TD) of −145.07 (95% confidence interval: −284.57, −5.56; *P* = .0416). In subgroup analyses, CATVS was significantly shorter for peramivir‐single vs oseltamivir among patients with influenza A (TD: −206.31 [−383.86, −28.76]; *P* = .0231), bronchial asthma (TD: −156.57 [−300.22, −12.92]; *P* = .0328), baseline respiratory severity score <5 (TD: −265.32 [−470.42, −60.21]; *P* = .0120), and age <65 (TD: −184.30 [−345.08, −23.52]; *P* = .0249).

**Conclusions:**

In patients with chronic respiratory diseases, peramivir‐single was not significantly different from peramivir‐repeat and was more effective than oseltamivir at alleviating respiratory symptoms.

## 1 INTRODUCTION

Influenza is a potentially life‐threatening illness associated with seasonal epidemics that result in significant societal disruption and morbidity.^1^, ^2^ Progression of infection to the lower respiratory tract can prove fatal, particularly in patients with chronic respiratory diseases such as bronchial asthma, chronic bronchitis, and chronic obstructive pulmonary disease (COPD).^3^, ^4^ Susceptible individuals have a high risk of acute respiratory distress syndrome, which is typically triggered by influenza A infection.^3^

Antiviral treatment with a neuraminidase inhibitor (NAI) can bring clinical benefits, including clearing virus, alleviating symptoms, reducing transmission,^5^ and potentially improving survival.^1^, ^6^ NAI efficacy has been explored predominantly in patients with uncomplicated seasonal influenza.^7^, ^8^, ^9^, ^10^, ^11^ Among these agents, intravenous peramivir, including a single‐dose 300 mg regimen, showed more rapid symptom alleviation compared with placebo^11^ and other NAIs.^8^, ^9^, ^10^ However, further data are needed for high‐risk patients with chronic respiratory diseases that can be aggravated by influenza, leading to delayed recovery from influenza symptoms.^12^, ^13^

A phase III trial previously investigated intravenous peramivir 300 or 600 mg/d for 1‐5 days as needed in high‐risk patients.^14^ The median duration of influenza illness was 114.4 and 42.3 hours in the 300 and 600 mg groups, respectively (hazard ratio: 0.497; 90% confidence interval [CI], 0.251‐0.984). In a post hoc analysis, the effect of peramivir on symptom alleviation was assessed using an index value for area under the curve (AUC) vs time based on the changing total scores of cough, sore throat, and nasal congestion (M. Kato, Y. Saisho, H. Tanaka, T. Bando, unpublished results). Peramivir 600 mg appeared to be more effective than peramivir 300 mg, with the former demonstrating a higher reduction from baseline in total symptoms at 2 weeks.

The primary objective of this study was to compare peramivir 600 mg repeat dose (1200 mg total dose) with peramivir 300 mg single dose and oseltamivir 75 mg twice daily in patients with influenza A or B infection and chronic respiratory diseases. The study also compared the effect of peramivir 300 mg single dose with oseltamivir. Secondary objectives reported here include changes in respiratory symptom scores over time, virus titer, and safety; additional outcomes will be reported separately.

## 2 METHODS

### 2.1 Study design

This was a 2‐week, multicenter, randomized, open‐label study to evaluate intravenous peramivir 600 mg repeat dose, intravenous peramivir 300 mg single dose, or oral oseltamivir 75 mg twice‐daily treatment in patients with confirmed influenza A or B together with concomitant bronchial asthma, COPD, or pulmonary fibrosis. The study was conducted between October 2017 and February 2019, encompassing two influenza seasons, across 50 sites in Japan. The study was conducted in accordance with the Declaration of Helsinki and, from October 2017 through December 2018, Ethical Guidelines for Medical and Health Research Involving Human Subjects. The study was a specified clinical trial as defined by the revised 2017 Clinical Trials Act and, therefore, from January 2019 through study completion, followed the guidelines set forth in the Act. The protocol was reviewed and approved by local ethical review boards and, in January 2019, by the clinical research board of Nippon Medical University, as per the Act. Patients gave written informed consent. The study was registered at the UMIN‐CTR Clinical Trials Registry (https://www.umin.ac.jp/ctr/index.htm, identifier: UMIN000030118) and at the Japan Registry of Clinical Trials (https://jrct.niph.go.jp/en-latest-detail/jRCTs031180322, identifier: jRCTs031180322).

**Figure 1:**
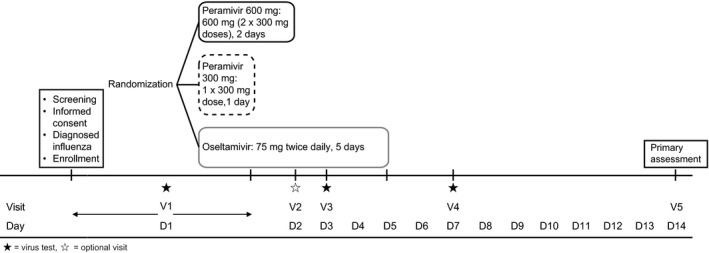
Study design. Screening visit (Day 1): patient consent, evaluation of patient demographics and clinical characteristics, physical examination, axillary body temperature, assessment of influenza symptoms, nasal cavity evaluation and throat swab, and confirmation of influenza using the rapid antigen test. Day 2: patients assigned to peramivir 600 mg received repeat treatment; if available, patients in any arm had physical examination and virus test. Day 3: physical examination, virus testing, and CAT, oxygen saturation, and respiratory function assessments in all patients, and clinical examination in patients in the two peramivir groups. Day 7: physical examination, virus testing, and testing for CAT, oxygen saturation, and respiratory function in all patients; clinical examination in patients in the oseltamivir arm. Day 14: physical examination, clinical examination (where possible), CAT, oxygen saturation, and respiratory function assessments in all patients. Patients were instructed to record their axillary body temperature four times per day on Days 1‐3 and twice per day from Day 4, and to record their influenza symptom scores twice per day on Days 1‐7, then once daily from Day 8. CAT, chronic obstructive pulmonary disease assessment test

Enrollment occurred within 48 hours from influenza onset defined as an initial ≥1°C increase in axillary body temperature above normal or worsening of ≥1 systemic or respiratory symptom compared with normal. All patients had ≥4 clinic visits (Figure 1). During a screening visit, influenza diagnosis was confirmed using the rapid antigen test. Patients were instructed in the use of a daily diary to record influenza symptom scores and temperature. A COPD assessment test (CAT) was conducted together with oxygen saturation and respiratory function testing. Patients were assigned to treatment, and the study drug was administered. Patients assigned to peramivir 600 mg repeat dose had an additional visit to receive the repeat treatment on Day 2. Adverse events (AEs) were monitored throughout the 14‐day study period.

### 2.2 Study population

Eligible patients were male or female inpatients or outpatients diagnosed with influenza aged 16‐79 years, with those aged 16‐19 years requiring consent from a legal guardian. Other key inclusion criteria were the following: a total symptom score for cough, sore throat, and nasal congestion of ≥3 including a score of ≥1 for cough, and ≥1 systemic symptom that scored ≥2 for headache, muscle or joint pain, heat or chills, or fatigue; nasal or throat swab with a positive rapid influenza test; maximum axillary temperature ≥37.5°C for ≥12 hours before screening; and receiving treatment for bronchial asthma, pulmonary fibrosis, or COPD. Key exclusion criteria were the following: concomitant infectious disease requiring treatment with a systemic antibacterial, antifungal, or antiviral drug; history of convulsions or other neurological symptoms within the past 2 years; chronic respiratory failure requiring management on a mechanical ventilator; diabetes with glycated hemoglobin A1c >10% within 4 weeks prior to screening; previous treatment with an NAI, amantadine hydrochloride, or baloxavir marboxil within the previous 7 days; cardiovascular disease requiring hospitalization, and other serious diseases requiring treatment, including congestive heart failure, central nervous system diseases, metabolic diseases, malignancies, renal dialysis, and transplantation within the previous 12 months.

### 2.3 Randomization and treatment

Patients were randomized (1:1:1) to peramivir 600 mg repeat dose administered as two 300 mg intravenous infusions on two consecutive days (ie, 1200 mg total dose), peramivir 300 mg single dose administered as a single 300 mg infusion, or oral oseltamivir 75 mg twice daily for 5 days (Figure 1). Infusion time was 15‐75 minutes for peramivir 600 mg repeat dose and 15‐45 minutes for peramivir 300 mg single dose. Randomization was conducted using the minimization method, stratified by total score of respiratory symptoms (≥5, <5) and underlying respiratory disease (bronchial asthma, COPD, or pulmonary fibrosis). Concomitant drugs (except topical medicines) such as antivirals, antifungals, antipyretics (except acetaminophen), general cold drugs, antihistaminic drugs, immunosuppressive drugs, Chinese medicine for influenza virus, and investigational drugs were not permitted. Patients could take a chemical mediator release inhibitor or leukotriene receptor antagonist as an alternative to antihistaminic drugs.

### 2.4 Outcome measures

The primary efficacy endpoint was “cumulative area of time vs symptoms” (CATVS) expressed as an index AUC of the total score of three respiratory symptoms (cough, sore throat, and nasal congestion) for 2 weeks (from Visit 1 [baseline] to Visit 5 [Day 14]). Influenza symptom severity was assessed using seven items including the three respiratory symptoms and four systemic symptoms (headache, muscle or joint pain, feverishness or chills, and fatigue) by patient diary. Symptom severity was scored as 0 (no symptoms), 1 (mild), 2 (moderate), or 3 (severe). Secondary efficacy endpoints were mean change from baseline over Visits 2‐5 in the total score of three respiratory symptoms, and mean change from baseline over Visits 2, 3, and 4 in virus titer, expressed as median 50% tissue culture infectious dose (TCID_50_) per mL. Nasal or throat swabs were sent to a central laboratory for viral titer measurement (LSI Medience Corporation). Safety assessments included the frequency of treatment‐emergent AEs (TEAEs), serious TEAEs (SAEs), and discontinuations due to TEAEs.

### 2.5 Statistical analysis

The planned sample size of 70 patients per treatment group was based on an estimate of 64 patients per group needed to provide 80% power to detect a difference between treatments with a two‐sided significance level of .05. Assumptions were further based on the results of a post hoc analysis of a phase III study of high‐risk patients,^14^ which showed a difference in index AUC for the total scores of cough, sore throat, and nasal congestion between peramivir 600 mg/d and peramivir 300 mg/d of 11.5; the standard deviation of each treatment group was 24.0. Oseltamivir was assumed to have the same effect on index AUC as peramivir 300 mg/d.

The primary efficacy analyses were conducted using the intent‐to‐treat (ITT) population, which included all randomized patients who received ≥1 dose of study drug and were eligible for efficacy analysis. The primary efficacy endpoint was analyzed using analysis of covariance with the weighted Holm method for multiplicity adjustment (with two‐sided significance level of .05 split into .04 and .01, respectively, for comparisons of peramivir 600 mg repeat dose with peramivir 300 mg single dose and with oseltamivir), AUC of the total score of three respiratory symptoms over 2 weeks as response variable, and total score at baseline and chronic respiratory disease as covariates. The comparison between peramivir 300 mg and oseltamivir was a secondary analysis. A subgroup analysis was conducted according to influenza virus type, chronic respiratory disease, severity of three respiratory symptoms (<5, ≥5), and age (<65 years, ≥65 years).

A secondary efficacy analysis of all pairwise comparisons was conducted. In the ITT population, the between‐group difference in the mean change from baseline in total score of the three respiratory symptoms every 24 hours was analyzed using a linear model with intra‐patient correlations between time points. The model included groups, time points, interaction between groups and time points, and chronic respiratory disease as explanatory variables with unstructured intra‐patient correlation. The degrees of freedom were adjusted using Kenward and Roger approximation. Safety analyses were conducted using the safety analysis set (SAS), which included all patients who received ≥1 dose of study drug. TEAEs were categorized by system organ class and preferred term (Medical Dictionary for Regulatory Activities, version 22.0). No multiplicity adjustment was conducted except for the primary efficacy analysis. Analyses were conducted using SAS software version 9.3 (SAS Institute).

## 3 RESULTS

### 3.1 Demographic and baseline clinical characteristics

Of 214 patients randomized, 209 received ≥1 dose of study drug and comprised the SAS (Figure S1). Screening data before obtaining consent were not available, but the major reason for patient ineligibility was not having a body temperature ≥37.5°C during the previous 12 hours. In the peramivir 600 mg repeat‐dose arm, one patient who withdrew consent was not included in the safety or ITT analyses. In the peramivir 300 mg single‐dose arm, four patients (two without written consent, one who withdrew consent, and one who required a prohibited drug) were not included in the SAS; in addition, one patient who received the allocated treatment was not included in the ITT population because they did not have a body temperature ≥37.5°C within 12 hours before screening and therefore did not meet this inclusion criterion. Patient demographics and baseline clinical characteristics were well balanced between treatment arms, with no significant differences (Table 1). Most patients were outpatients, aged <65 years, never smokers, with comorbid bronchial asthma, a total score of three respiratory symptoms ≥5, and a predominance of infection by influenza A.

**Table 1:** Baseline demographics and disease characteristics (safety analysis set)

Characteristic	Peramivir 600 mg N = 70	Peramivir 300 mg N = 67	Oseltamivir N = 72	*P*‐value
Age				
<65 y	51 (72.9)	54 (80.6)	56 (77.8)	.5747^a^
≥65 y	19 (27.1)	13 (19.4)	16 (22.2)	
Sex				
Male	35 (50.0)	30 (44.8)	28 (38.9)	.4030^a^
Female	35 (50.0)	37 (55.2)	44 (61.1)	
Smoking status				
Never	46 (65.7)	43 (64.2)	45 (62.5)	.9414^b^
Former smoker	15 (21.4)	13 (19.4)	23 (31.9)	
Current smoker	9 (12.9)	11 (16.4)	4 (5.6)	
Hospitalization				
Inpatient	3 (4.3)	7 (10.4)	2 (2.8)	.1508^a^
Outpatient	67 (95.7)	60 (89.6)	70 (97.2)	
Type of influenza				
A virus	42 (60.0)	42 (62.7)	46 (63.9)	.9049^a^
B virus	28 (40.0)	25 (37.3)	26 (36.1)	
Influenza time of onset				
≤12 h	15 (21.4)	10 (14.9)	9 (12.5)	.5892^b^
>12 to ≤24 h	29 (41.4)	27 (40.3)	32 (44.4)	
>24 to ≤36 h	9 (12.9)	12 (17.9)	17 (23.6)	
>36 to ≤48 h	15 (21.4)	16 (23.9)	9 (12.5)	
>48 h	1 (1.4)	1 (1.5)	2 (2.8)	
Unknown	1 (1.4)	1 (1.5)	3 (4.2)	
Chronic respiratory disease				
COPD	5 (7.1)	4 (6.0)	6 (8.3)	.9903^c^
Bronchial asthma	64 (91.4)	62 (92.5)	65 (90.3)	
Pulmonary fibrosis	1 (1.4)	1 (1.5)	1 (1.4)	
Total score of three respiratory symptoms				
≥5 score	45 (64.3)	44 (65.7)	44 (61.1)	.8443^a^
<5 score	25 (35.7)	23 (34.3)	28 (38.9)	
Body temperature				
<37°C	0 (0.0)	0 (0.0)	0 (0.0)	.1503^b^
≥37°C to <38°C	21 (30.0)	30 (44.8)	33 (45.8)	
≥38°C to <39°C	37 (52.9)	30 (44.8)	26 (36.1)	
≥39°C to <40°C	10 (14.3)	7 (10.4)	11 (15.3)	
≥40°C	2 (2.9)	0 (0.0)	2 (2.8)	
Influenza vaccine in the past 6 mo				
No	44 (62.9)	43 (64.2)	43 (59.7)	.8609^a^
Yes	26 (37.1)	24 (35.8)	29 (40.3)	
History of influenza ever				
No	62 (88.6)	63 (94.0)	69 (95.8)	.2746^a^
Yes	8 (11.4)	4 (6.0)	3 (4.2)	
Concomitant illness				
Pneumonia	0 (0.0)	2 (3.0)	2 (2.8)	.4688^a^
Bronchitis	0 (0.0)	2 (3.0)	1 (1.4)	.3175^a^
Otitis media	0 (0.0)	0 (0.0)	0 (0.0)	‐
Sinusitis	2 (2.9)	3 (4.5)	3 (4.2)	.9084^a^
Prior non‐drug treatment^d^				
No	54 (77.1)	54 (80.6)	56 (77.8)	.9115^a^
Yes	16 (22.9)	13 (19.4)	16 (22.2)	
Prior drugs^d^				
No	3 (4.3)	4 (6.0)	4 (5.6)	.9291^a^
Yes	67 (95.7)	63 (94.0)	68 (94.4)	

Note

All values are n (%).

COPD, chronic obstructive pulmonary disease.

^a^ Fisher’s exact test.

^b^ Kruskal‐Wallis test.

^c^ Pearson’s chi‐squared test.

^d^ For influenza or chronic respiratory disease.

### 3.2 Primary outcome measure

#### 3.2.1 Peramivir 600 mg repeat‐dose vs peramivir 300 mg single‐dose treatment (primary analysis)

There was no difference between peramivir 600 mg repeat dose and 300 mg single dose with respect to the primary outcome of CATVS (Table 2). The mean index AUC of 782.78 for peramivir 600 mg repeat dose equated to an estimated between‐group treatment difference (TD) relative to peramivir 300 mg single dose of 66.70 (95% CI: −73.62, 207.02; *P* = .4371). Similarly, there was no difference between peramivir 600 mg repeat dose and oseltamivir, with an estimated between‐group TD of −78.36 (95% CI: −215.69, 58.96; *P* = 1.0000).

**Table 2:** Cumulative area of time vs symptoms expressed as an index value for area under the curve of the total score of cough, sore throat, and nasal congestion from the start of study drug administration to 2 wk post‐administration (ITT population)

Variable	n	Mean	SD	Min	Median	Max
Peramivir 600 mg	70	782.78	487.17	64.4	771.60	2296.3
Peramivir 300 mg	66	717.35	347.55	47.8	684.49	1625.8
Oseltamivir	72	856.34	404.99	85.7	859.32	1856.4

	Estimated difference between 2 groups	SE	95% CI	*P*‐value
Peramivir 600 mg vs peramivir 300 mg	66.70	71.16	−73.62, 207.02	.4371^a^
Peramivir 600 mg vs oseltamivir	−78.36	69.64	−215.69, 58.96	1.0000^a^
Peramivir 300 mg vs oseltamivir	−145.07	70.75	−284.57, −5.56	.0416^b^

CI, confidence interval; ITT, intent‐to‐treat; Max, maximum; Min, minimum; SD, standard deviation; SE, standard error.

^a^ Adjusted *P*‐value by weighted Holm method.

^b^ Non‐adjusted *P*‐value.

#### 3.2.2 Peramivir 300 mg single‐dose vs oseltamivir treatment

Cumulative area of time vs symptoms was significantly shorter for peramivir 300 mg single dose compared with oseltamivir (Table 2). The mean index AUC of 717.35 for peramivir 300 mg equated to an estimated between‐group TD relative to oseltamivir of −145.07 (95% CI: −284.57, −5.56; *P* = .0416), indicating shorter time to symptom resolution.

#### 3.2.3 Subgroup analyses

Compared with peramivir 600 mg repeat dose or oseltamivir, treatment with peramivir 300 mg single dose was associated with shorter CATVS across a range of subgroups, including virus type, those with bronchial asthma or pulmonary fibrosis, symptom severity score <5 or ≥5, and age <65 years (Table S1). The estimated TD was significant for the comparison between peramivir 300 mg single dose and oseltamivir among patients with influenza A (TD: −206.31; 95% CI: −383.86, −28.76; *P* = .0231), but not for patients with influenza B where CATVS was similar for all three arms. The estimated TD was also significant for the comparison between peramivir 300 mg single dose and oseltamivir among patients with bronchial asthma (TD: −156.57; 95% CI: −300.22, −12.92; *P* = .0328), those with a baseline total respiratory symptom severity score <5 (TD: −265.32; 95% CI: −470.42, −60.21; *P* = .0120), and for patients <65 years old (TD: −184.30; 95% CI: −345.08, −23.52; *P* = .0249). In each case, the TD indicated a shorter CATVS for peramivir 300 mg single dose. Additionally, the estimated TD was significant for the comparison between peramivir 600 mg repeat dose and oseltamivir for patients with a baseline total respiratory symptom score <5 (TD: −261.22; 95% CI: −459.30, −63.15; *P* = .0105).

### 3.3 Secondary outcome measures

#### 3.3.1 Changes in symptoms

**Figure 2:**
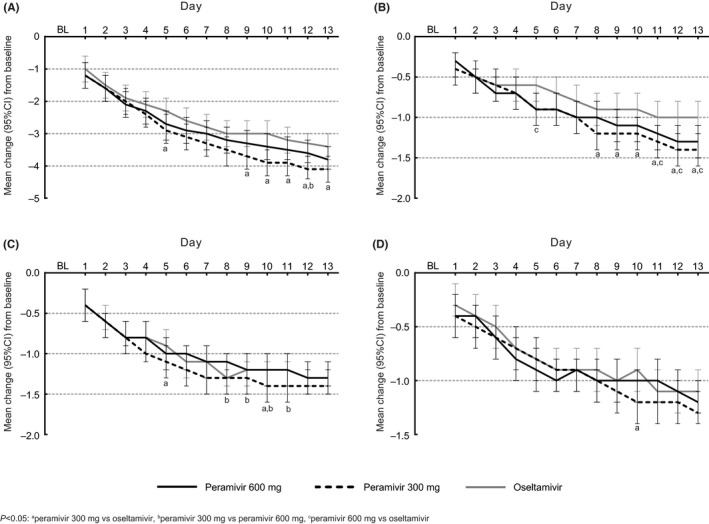
Overall mean change from baseline in score of three respiratory symptoms to Day 13: (A) total score, (B) cough, (C) sore throat, and (D) nasal congestion. Values are mean and 95% CI (ITT population). BL, baseline; CI, confidence interval; ITT, intent‐to‐treat. ^a^ *P* < 0.05 (peramivir 300 mg vs oseltamivir). ^b^ *P* < 0.05 (peramivir 300 mg vs peramivir 600 mg). ^c^ *P* < 0.05 (peramivir 600 mg vs oseltamivir)

Peramivir 300 mg single dose was associated with significantly greater decreases from baseline in total symptom score compared with both oseltamivir (Day 5 and Days 9‐13) and peramivir 600 mg repeat dose (Day 12) (Figure 2A). Compared with oseltamivir, cough scores decreased significantly more with both peramivir 300 mg single dose (Days 8‐13) and peramivir 600 mg repeat dose (Day 5 and Days 11‐13) (Figure 2B). Decreases in sore throat scores were significantly greater with peramivir 300 mg single dose than with oseltamivir (Days 5 and 10) and peramivir 600 mg repeat dose (Days 8‐11) (Figure 2C). Decreases in nasal congestion score were similar in the three groups, except for a greater decrease with peramivir 300 mg single dose compared with oseltamivir on Day 10 (Figure 2D).

#### 3.3.2 Virus titer

**Figure 3:**
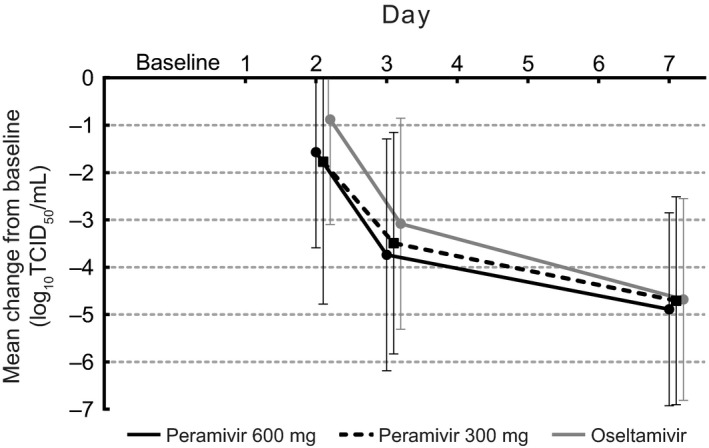
Overall change from baseline of virus titer at Days 2, 3, and 7 (ITT population). Values are mean and SD. Mean (SD) baseline values were 5.63 (2.04), 5.38 (2.21), and 5.44 (2.13) log_10_TCID_50_/mL in the peramivir 600 mg, peramivir 300 mg, and oseltamivir groups, respectively. ITT, intent‐to‐treat; SD, standard deviation; TCID_50_, 50% tissue culture infectious dose

The reduction in symptom score was associated with a decrease in viral titer at Days 2, 3, and 7 (Figure 3). At Day 3 following completion of dosing in the two peramivir arms but not oseltamivir, the mean (standard deviation) reduction from baseline in virus titer (expressed as log_10_TCID_50_/mL) was −3.74 (2.45) for peramivir 600 mg repeat dose, −3.49 (2.34) for peramivir 300 mg single dose, and −3.08 (2.23) for oseltamivir.

In a subanalysis of viral titer by influenza type, patients with influenza A had a more rapid reduction in viral titer vs those with influenza B (data not shown). Among patients with influenza A, peramivir 300 mg single dose compared with peramivir 600 mg repeat dose was associated with a significantly greater reduction in viral titer at Day 2 (*P* = .0268), and peramivir 600 mg repeat dose compared with oseltamivir was associated with a significantly greater reduction in viral titer at Day 3 (*P* = .0313). There were no differences in viral titer between the three arms at Day 7.

### 3.4 Safety and tolerability measures

Treatment with peramivir 600 mg repeat dose, peramivir 300 mg single dose, or oseltamivir was well tolerated (Table S2). The incidence of any TEAEs was higher among patients treated with peramivir 600 mg repeat dose (25.7%) compared with either peramivir 300 mg single dose (13.4%) or oseltamivir (13.9%). However, the only TEAEs that occurred in ≥2 patients in any arm were diarrhea, hepatic function abnormal, vomiting, and decreased appetite. Three patients experienced SAEs: one patient each with vomiting and pneumonia in the peramivir 600 mg repeat‐dose arm, and one patient with pneumococcal pneumonia in the peramivir 300 mg single‐dose arm.

## 4 DISCUSSION

This is the first prospective, randomized, head‐to‐head study of oseltamivir and peramivir 600 mg repeat‐dose and 300 mg single‐dose regimens in high‐risk patients with chronic respiratory diseases. The findings showed that in patients with respiratory diseases, predominantly bronchial asthma, there was no difference between the peramivir dose arms in CATVS. However, treatment with peramivir 300 mg single dose compared with oseltamivir was associated with a significant reduction in CATVS, suggestive of a shorter cumulative time with symptoms for patients treated with single‐dose peramivir. Considering individual respiratory symptoms, the reduction from baseline in cough symptom score was significantly greater for patients treated with peramivir 300 mg single dose or peramivir 600 mg repeat dose compared with oseltamivir. Among patients with influenza A, peramivir 300 mg single dose was associated with a shorter CATVS than oseltamivir. Compared with oseltamivir, peramivir 300 mg single dose was also associated with a shorter time to resolution of respiratory symptoms for patients with bronchial asthma, those aged <65 years, and patients with a baseline total respiratory symptom score of <5. Further, NAI treatment was well tolerated irrespective of treatment. Collectively, these findings suggest that peramivir 300 mg single dose is effective and well tolerated in high‐risk patients with chronic respiratory diseases and is able to reduce the duration of influenza symptoms compared with oseltamivir. These results also support previous evidence that, owing to the rapid increase in plasma concentration after administration,^15^ peramivir reduces virus levels more quickly than oseltamivir^8^ and independently of immune status.^6^

Previous head‐to‐head trials of NAIs have focused on the general patient population receiving treatment for acute uncomplicated seasonal influenza.^7^, ^8^, ^16^, ^17^ Studies of patients with high‐risk features have also been conducted,^14^, ^18^, ^19^, ^20^, ^21^, ^22^ including with peramivir 300 or 600 mg/d administered for 1‐5 days (mostly 1‐2 days) as needed.^14^ This latter trial included patients with diabetes and chronic respiratory diseases and showed a shorter median duration of influenza for patients who received peramivir 600 mg/d compared with 300 mg/d.^14^ However, the sample size was small. Another study directly compared intravenous peramivir 600 mg single dose (a second dose was necessary in three of 46 patients) with oseltamivir 75 mg twice daily for 5 days in high‐risk patients with influenza A or B infection.^19^ Changes in mean total symptom scores and virus titer were similar between treatments, whereas peramivir 600 mg single dose was somewhat better tolerated. The present study adds to these findings in that it establishes either peramivir 300 mg single dose or 600 mg repeat dose as an effective antiviral option in patients with chronic respiratory diseases. In particular, peramivir 300 mg single dose offered greater efficacy than oseltamivir in patients with bronchial asthma and influenza A. Although a potential benefit for the 600 mg repeat‐dose regimen could not be established, with the 300 mg single‐dose regimen providing significant antiviral effect and symptom reduction, the former regimen may be more appropriate for the inpatient setting. Thus, it is relevant that the majority of patients treated in this study were outpatients. Our findings also confirm the safety of intravenous peramivir in patients with high‐risk features, with overall safety consistent with post‐marketing safety evaluations of peramivir.^18^

In addition to providing superior symptom relief overall, peramivir 300 mg single dose also had an impact on cough. Influenza symptoms are triggered in response to upper airway infection, damage to the respiratory epithelium, and the subsequent host immune response.^23^ Peramivir’s mechanism of action is explained by its potent inhibition of influenza neuraminidase enzyme, with prolonged binding compared with either oseltamivir or zanamivir.^24^ Given peramivir’s effect on cough was superior to oseltamivir, this indicates that its strong antiviral effect may reduce damage to the airway epithelium leading to earlier alleviation of symptoms. Viruses such as influenza are implicated in the majority of asthma and COPD exacerbations.^25^ The diminished cough associated with peramivir in the present context may reflect reductions in virus‐associated epithelial activation and degeneration, which stimulate persistent cough through mechanisms involving inflammatory mediators^26^ and stimulation of C‐fibers,^27^ respectively.

### 4.1 Study strengths and limitations

This study permitted robust comparison between peramivir and oseltamivir while minimizing the potential for selection bias through randomization. The inclusion of high‐risk patients, for whom NAI head‐to‐head data are limited, expanded the understanding of peramivir’s efficacy beyond patients with uncomplicated influenza to include high‐risk patients who are likely to benefit most from NAI treatment. As an open‐label study, there was the potential for selection bias related to the inability to conceal treatment allocation. Blinded outcome assessment was not undertaken as it would have required a double‐blind, double‐dummy design. The inclusion of a control group through which to compare treatment outcomes in patients with and without chronic respiratory diseases would have strengthened the study. Outcome assessment depended on subjective self‐reports of respiratory symptoms, which may have resulted in detection bias as patients receiving in‐clinic intravenous treatment may have viewed symptom resolution more positively than patients taking oral treatment. The peramivir 600 mg repeat‐dose regimen required a clinic visit on the second day, which potentially affected patients’ subsequent recovery. Regardless, intravenous peramivir even for the 600 mg repeat‐dose regimen achieved at least comparable results to oseltamivir and showed superiority in some measures, suggesting that this was not a study limitation.

## 5 CONCLUSION

In the main analysis, there were no significant differences in CATVS between peramivir 600 mg repeat dose and either peramivir 300 mg single dose or oseltamivir. Secondary analysis showed a significant difference between peramivir 300 mg single dose and oseltamivir. Significant differences between peramivir and oseltamivir were seen for several secondary endpoints, including changes in respiratory symptoms (especially cough). Differential effects of peramivir and oseltamivir on other outcomes, including the COPD Assessment Test, will be reported elsewhere.

## CONFLICT OF INTEREST

MK is a steering committee member for AstraZeneca K.K., Nippon Boehringer Ingelheim Co., Ltd, GlaxoSmithKline K.K., and Shionogi & Co., Ltd., and has given lectures for AstraZeneca K.K., Nippon Boehringer Ingelheim Co., Ltd., GlaxoSmithKline K.K., Novartis Pharma K.K., Sanofi K.K., and Shionogi & Co., Ltd. YS is an employee of Shionogi & Co., Ltd. HT has received speaker honoraria from GlaxoSmithKline K.K., Kyorin Pharmaceutical Co., Ltd., AstraZeneca K.K., Shionogi & Co., Ltd., Meiji Seika Pharma Co., Ltd., Nippon Boehringer Ingelheim Co., Ltd., Sanofi K.K., Novartis Pharma K.K., Hisamitsu Pharmaceutical Co., Inc., and TEIJIN Pharma Limited. TB is an executive director of Japan Physicians Association and has received study funding support from Shionogi & Co., Ltd. and Daiichi Sankyo Co., Ltd.

## AUTHOR CONTRIBUTIONS

MK: Study design; principal investigator. YS: Study design; statistical analysis (interpretation of analyses conducted by EP‐CRSU Co., Ltd). HT: Data collection; co‐investigator. TB: Data collection; co‐investigator. All authors participated in the interpretation of study results, and in the drafting, critical revision, and approval of the final version of the manuscript.

## Supporting information

Fig S1

Table S1

Table S2
