# Supplementary figures and images for: Effect of peramivir on respiratory symptom improvement in patients with influenza virus infection and pre‐existing chronic respiratory disease: Findings of a randomized, open‐label study
Source: Influenza Other Respir Viruses. 2020 Jul 17;15(1):132–41. doi: 10.1111/irv.12788 (PMC7767948; doi:10.1111/irv.12788)

**Supplementary Figure 1** Patient disposition.


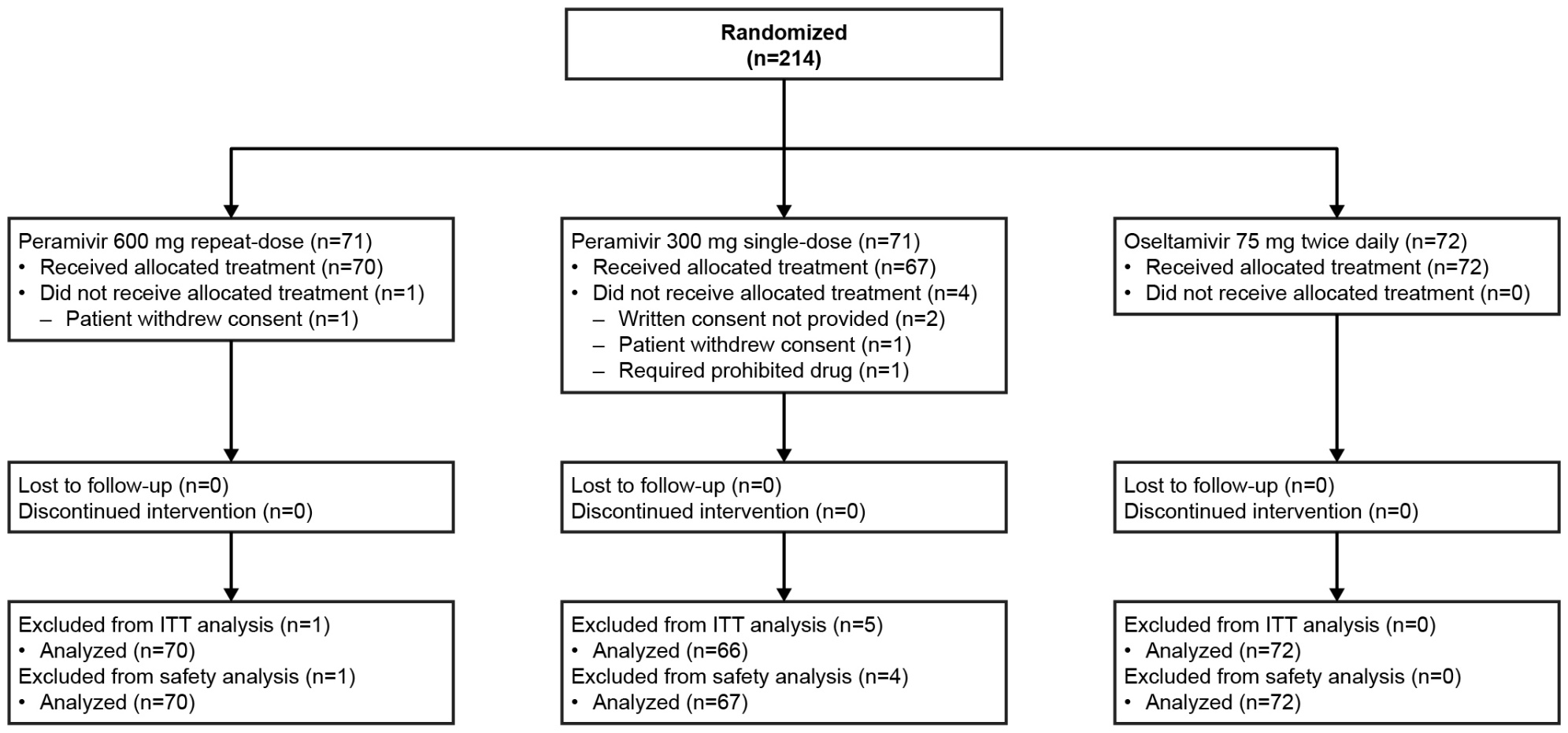


Abbreviation: ITT, intent-to-treat.

Supplement: Supplementary file 1 — Fig S1 [file IRV-15-132-s001.docx]
