# Supplementary material for: Effect of peramivir on respiratory symptom improvement in patients with influenza virus infection and pre‐existing chronic respiratory disease: Findings of a randomized, open‐label study
Source: Influenza Other Respir Viruses. 2020 Jul 17;15(1):132–41. doi: 10.1111/irv.12788 (PMC7767948; doi:10.1111/irv.12788)
Supplement: Supplementary file 2 — Table S1 [file IRV-15-132-s002.docx]

**Supplementary Table 1** Cumulative area of time vs symptoms expressed as an index value for area under the curve of the total score of cough, sore throat, and nasal congestion from the start of study drug administration to 2 weeks post-administration: subgroup analysis (ITT population)

| **Subgroup** |  | **Peramivir**  **600 mg** | **Peramivir**  **300 mg** | **Oseltamivir** |
| --- | --- | --- | --- | --- |
| Type of influenza |  |  |  |  |
| Virus A | n | 42 | 42 | 46 |
|  | Mean ± SD | 741.42 ± 493.28 | 654.74 ± 339.14 | 856.97 ± 433.27 |
| Virus B | n | 28 | 24 | 26 |
|  | Mean ± SD | 844.81 ± 479.96 | 826.90 ± 341.54 | 855.24 ± 357.64 |
| Chronic respiratory disease | |  |  |  |
| COPD | n  Mean ± SD | 5  681.52 ± 502.38 | 4  806.17 ± 443.85 | 6  833.94 ± 524.66 |
| Bronchial asthma | n  Mean ± SD | 64  780.12 ± 485.62 | 61  716.10 ± 345.22 | 65  863.16 ± 398.58 |
| Pulmonary fibrosis | n  Mean | 1  1459.24 | 1  438.27 | 1  547.92 |
| Severity: total score of 3 respiratory symptoms | | |  |  |
| ≥5 score | n  Mean ± SD | 45  883.40 ± 511.01 | 43  786.88 ± 341.22 | 44  853.15 ± 437.90 |
| <5 score | n  Mean ± SD | 25  601.65 ± 387.86 | 23  587.35 ± 327.84 | 28  861.36 ± 354.78 |
| Age |  |  |  |  |
| <65 years | n  Mean ± SD | 51  798.52 ± 498.80 | 53  710.43 ± 363.99 | 56  871.65 ± 421.92 |
| ≥65 years | n  Mean ± SD | 19  740.50 ± 464.84 | 13  745.54 ± 281.21 | 16  802.78 ± 345.94 |

Abbreviations: COPD, chronic obstructive pulmonary disease; ITT, intent-to-treat; SD, standard deviation.
