# Supplementary material for: Effect of peramivir on respiratory symptom improvement in patients with influenza virus infection and pre‐existing chronic respiratory disease: Findings of a randomized, open‐label study
Source: Influenza Other Respir Viruses. 2020 Jul 17;15(1):132–41. doi: 10.1111/irv.12788 (PMC7767948; doi:10.1111/irv.12788)
Supplement: Supplementary file 3 — Table S2 [file IRV-15-132-s003.docx]

**Supplementary Table 2** The incidence of treatment-emergent adverse events (Safety analysis set)

| **Preferred term^†^, n (%)** | **Peramivir**  **600 mg**  **N=70** | **Peramivir**  **300 mg**  **N=67** | **Oseltamivir**  **N=72** |
| --- | --- | --- | --- |
| Total | 18 (25.7) | 9 (13.4) | 10 (13.9) |
| Diarrhea | 4 (5.7) | 1 (1.5) | 2 (2.8) |
| Hepatic function abnormal | 1 (1.4) | 2 (3.0) | 1 (1.4) |
| Vomiting | 2 (2.9) | 0 (0.0) | 1 (1.4) |
| Decreased appetite | 2 (2.9) | 0 (0.0) | 0 (0.0) |
| Nausea | 1 (1.4) | 0 (0.0) | 1 (1.4) |
| Abdominal pain | 1 (1.4) | 0 (0.0) | 0 (0.0) |
| Loose stools | 1 (1.4) | 0 (0.0) | 0 (0.0) |
| Gastroenteritis | 1 (1.4) | 0 (0.0) | 0 (0.0) |
| Nasopharyngitis | 1 (1.4) | 0 (0.0) | 0 (0.0) |
| Pneumonia | 1 (1.4) | 0 (0.0) | 0 (0.0) |
| Spinal compression fracture | 1 (1.4) | 0 (0.0) | 0 (0.0) |
| Blood creatine phosphokinase increased | 1 (1.4) | 0 (0.0) | 0 (0.0) |
| Urticaria | 1 (1.4) | 0 (0.0) | 0 (0.0) |
| Neurogenic shock | 1 (1.4) | 0 (0.0) | 0 (0.0) |
| White blood cell count decreased | 1 (1.4) | 0 (0.0) | 0 (0.0) |
| Leukopenia | 0 (0.0) | 1 (1.5) | 0 (0.0) |
| Chest pain | 0 (0.0) | 1 (1.5) | 0 (0.0) |
| Herpes simplex | 0 (0.0) | 1 (1.5) | 0 (0.0) |
| Pneumococcal pneumonia | 0 (0.0) | 1 (1.5) | 0 (0.0) |
| Syncope | 0 (0.0) | 1 (1.5) | 0 (0.0) |
| Allergic dermatitis | 0 (0.0) | 1 (1.5) | 0 (0.0) |
| Vertigo | 0 (0.0) | 0 (0.0) | 1 (1.4) |
| Visual impairment | 0 (0.0) | 0 (0.0) | 1 (1.4) |
| Upper abdominal pain | 0 (0.0) | 0 (0.0) | 1 (1.4) |
| Enterocolitis | 0 (0.0) | 0 (0.0) | 1 (1.4) |
| Oral herpes | 0 (0.0) | 0 (0.0) | 1 (1.4) |
| Laboratory test abnormal | 0 (0.0) | 0 (0.0) | 1 (1.4) |
| White blood cell analysis abnormal | 0 (0.0) | 0 (0.0) | 1 (1.4) |
| Erythema | 0 (0.0) | 0 (0.0) | 1 (1.4) |
| Pruritus | 0 (0.0) | 0 (0.0) | 1 (1.4) |

†Adverse events were coded using Medical Dictionary for Regulatory Activities, version 22.0.
